# Neuroprotective Effects of a Cholecystokinin Analogue in the 1-Methyl-4-Phenyl-1,2,3,6-Tetrahydropyridine Parkinson’s Disease Mouse Model

**DOI:** 10.3389/fnins.2022.814430

**Published:** 2022-03-15

**Authors:** Zijuan Zhang, Hai Li, Yunfang Su, Jinlian Ma, Ye Yuan, Ziyang Yu, Ming Shi, Simai Shao, Zhenqiang Zhang, Christian Hölscher

**Affiliations:** ^1^Academy of Chinese Medical Sciences, Henan University of Chinese Medicine, Zhengzhou, China; ^2^School of Medical Sciences, Henan University of Chinese Medicine, Zhengzhou, China; ^3^Neurology Department of the Second Associated Hospital of Shanxi Medical University, Taiyuan, China

**Keywords:** Parkinson’s disease, CCK, insulin, MPTP, neuroinflammation, apoptosis, autophagy

## Abstract

Parkinson’s disease (PD) is a chronic neurodegenerative disease. Type 2 diabetes mellitus (T2DM) has been identified as a risk factor for PD. Drugs originally developed for T2DM treatment such as liraglutide have shown neuroprotective effects in mouse models of PD. Cholecystokinin (CCK) is a peptide hormone with growth factor properties. Here, we demonstrate the neuroprotective effects of the (pGLu)-(Gln)-CCK8 analogue in an acute PD mouse model induced by 1-Methyl-4-phenyl-1,2,3,6-tetrahydropyridine (MPTP). Administration of CCK analogue (50 nmol/kg ip.) for 14 days treatment improved the locomotor and exploratory activity of mice, and improved bradykinesia and movement balance of mice. The CCK analogue administration also restored tyrosine hydroxylase (TH) positive dopaminergic neurons number and synapse number (synaptophysin levels) in the substantia nigra pars compacta (SNpc). The CCK analogue decreased glia activation and neuroinflammation in the SNpc, and regulated autophagy dysfunction induced by MPTP. CCK analogue protected against mitochondrial damage and ER stress, and also decreased the ratio of apoptosis signaling molecules Bax/Bcl-2. Importantly, the CCK analogue improved the decrease of p-CREBS^133^ growth factor signaling in the SNpc. Therefore, the CCK analogue promotes cell survival of dopaminergic neuron in the SNpc by activating the cAMP/PKA/CREB pathway that also inhibits apoptosis and regulates autophagy impairment. The present results indicate that CCK analogue shows a promising potential for the treatment of PD.

## Introduction

Parkinson’s disease (PD) is a chronic degenerative disease of the central nervous system with a prevalence rate of 1.7% in people over 65 years and as high as 4–5% in people over 85 years (Kowal et al., 2013). The typical pathological manifestations of PD include a progressive loss and death of dopaminergic neurons in the substantia nigra pars compacta (SNpc), which leads to muscular rigidity, tremors, bradykinesia, and postural and gait abnormalities (Lang and Espay, 2018). Currently, no drugs are on the market that are disease modifying.

Recently, type 2 diabetes mellitus (T2DM) has been identified as a risk factor for PD (Sergi et al., 2019). It appears that insulin loses its function in the brains of PD patients which then impairs neuronal repair and survival. Glucagon-like peptide 1 (GLP-1) analogues that initially had been developed as drugs to treat diabetes have shown good neuroprotective effects in animal models of PD (Li et al., 2016; Hölscher, 2020; Zhang and Holscher, 2020). GLP-1 mimetics, e.g., liraglutide, exenatide, or lixisenatide, showed neuroprotective properties in the 1-Methyl-4-phenyl-1,2,3,6-tetrahydropyridine (MPTP) mouse model (Li et al., 2009; Liu et al., 2015; Yuan et al., 2017; Chen et al., 2018; Lin et al., 2021), and liraglutide is currently being tested in a phase II trial in PD patients (clinical trial identifier NCT02953665). Importantly, the GLP-1 receptor agonist exendin-4 showed good protective effects in a pilot clinical trial and a phase II clinical trial in PD patients (viles-Olmos et al., 2013, 2014; Athauda et al., 2017, 2019). These results inspired us to examine the potential neuroprotective effects of cholecystokinin (CCK) (Irwin et al., 2012) and chose the GLP-1 analogue liraglutide as a positive control. CCK belongs to the same peptide hormone family that modulates metabolism as insulin, GLP-1 and GIP do. CCK is widely expressed in the brain and is also a growth factor, a neurotransmitter in the brain and in peripheral neurons (Rehfeld, 2017). CCK is expressed in the highest concentrations in neocortical neurons (Fallon and Seroogy, 1985). There are two forms of the receptor, CCKR-1 and CCKR-2. The CCKR-1 is predominately expressed in the periphery and is activated by longer CCK fragments such as CCK-22 and 33. CCKR-2 is predominately expressed in the brain and is activated by short fragments such as CCK-8 (Lee et al., 2011). GLP-1, GIP and CCK activate G-protein linked membrane standing receptors that enhance cAMP levels (Irwin et al., 2013; Rehfeld, 2017) and activate the PLC-PKC cell signaling pathway (Estall and Drucker, 2003; Lee et al., 2011). This pathway can modulate intracellular calcium levels and furthermore modulate the release of neurotransmitters and gene expression (Lee et al., 2011).

A long-lasting protease-resistant analogue of CCK has been developed and tested in animal models of diabetes (Irwin et al., 2012, 2015). In addition, It has been reported that intraperitoneal (i.p.) injection of cholecystokinin (CCK-8) can stimulate the synthesis of Nerve Growth factor (NGF) in the brain and surrounding organs by activating the CCK receptor, thus playing a neuroprotective role (Tirassa et al., 2002). Crucially, CCK receptors are also found on dopaminergic neurons in the substantia nigra (Fallon and Seroogy, 1985), a neuronal population that is affected in Parkinson’s disease. CCK furthermore plays a role in memory formation and in synaptic transmission and plasticity (Chen et al., 2019). Until now, CCK receptor agonists have not been tested in animal models of neurodegenerative disorders.

We therefore tested the effects of a CCK analogue in the MPTP mouse model of PD to investigate their neuroprotective properties.

## Materials and Methods

### Peptides and Chemicals

The peptides (CCK analogue and Liraglutide) were synthetized by ChinaPeptides Co., Ltd., (Shanghai, China) with 95% purity. CCK analogue carboxyfluorescein (CCK-AC) was synthetized by JPT Peptide Technologies GmbH (Berlin, Germany). The purity of the peptide was analyzed by reversed-phase high HPLC and characterized using matrix-assisted laser desorption/ionisation time of flight (MALDI-TOF) mass spectrometry. MPTP were purchased from Solarbio Science & Technology Co., Ltd., (Beijing, China). Other chemicals used were of the highest quality commercially available.

Cholecystokinin analogue peptide sequence (Irwin et al., 2012; Rehfeld, 2017): H-pEQDYTGWMDF-NH2.

CCK analogue carboxyfluorescein sequence: Pyr-EQDYT GWMDF-Lys[(5(6)-Carboxyfluorescein)-NH2].

### Assessment of Cholecystokinin Analogue for Permeability of the Blood–Brain Barrier

The male C57BL/6 mice (25–30 g) were used to assess to the BBB permeability of CCK-AC. Fluorescence-labeled peptides can be used to test the BBB permeability, see Secher et al. (2014), Salinas et al. (2018) and Gabery et al. (2020) for details. Animals were randomly assigned to two groups (*n* = 4 per group): (1) control group treated with saline, (2) CCK-AC group treated with CCK-AC. CCK-AC were injected at 50 nmol/kg ip. The control group was administered with the equal volumes of the vehicle solution. 2 h later, all the animals were transcardially perfused with 0.9% NaCl and followed by 4% paraformaldehyde under anesthesia with isoflurane. The frozen brains were cut at 20 μm using a vibratome (Leica Microsystems, Wetzlar, Germany). Images were acquired using an Olympus light microscope. Fluorescence was quantified using Image-Pro Plus 6.0 by an experimenter blind to all groups.

### Animals

All the male C57BL/6 mice (2-month-old, 25–30 g) were obtained from the Hunan SJA Laboratary Animal Co., Ltd., All mice were housed in groups of three to five on a standard environment [temperature (22 + 2)°C; humidity 55%; 12 h light-dark cycle]. All experiments were conducted during the light cycle. Food and water was provided *ad libitum*, and were acclimatized for at least 1 week prior to the MPTP procedure. All procedures involved in this study was approved by the Animal Care and Use Committee of Henan University of Chinese Medicine (No: DWLL201903076). Animals were randomly assigned to the following groups (*n* = 12 per group): (1) control group treated with normal saline (NS) alone, (2) MPTP group treated with MPTP alone, (3) MPTP + CCK group treated with CCK analogue in combination with MPTP, (4) MPTP + liraglutide group treated with liraglutide in combination with MPTP. MPTP and drugs (CCK analogue and liraglutide) were dissolved in saline. Mice received 20 mg/kg/day MPTP in saline i.p. for seven consecutive days, a dose that previously showed good effects (Li et al., 2016; Zhang et al., 2020; Lv et al., 2021). Simultaneously, starting on the first day of MPTP injections, the CCK analogue and liraglutide were administered for 14 consecutive days injection once-daily. The time interval between two drugs administration was 2 h. The drug concentrations were: CCK analogue: 50 nmol/kg i.p., liraglutide: 50 nmol/kg i.p. To be able to compare the drugs directly, the same dose was chosen for both drugs. This dose chosen for Liraglutide showed good neuroprotective effects in previous studies (Liu et al., 2015). Control mice received equal volumes of the vehicle solution.

### Behavioral Tests

All mice of each group were evaluated in behavioral tests.

#### Open-Field Test

To evaluate locomotor and exploratory activity of mice in new environments, the open-field test was conducted after MPTP treatment. First, mice were placed in the middle of the test chamber to adapt to the activity for 10 min, and then the amount of time an animal stayed in a central area (Time in central zone%), the crossing line and rearings of the hind feet were recorded by the open-field analysis system within 5 min (Smart3.0, United States). After each animal was tested, the animal feces at the bottom of the chamber were cleaned and the chambers were dried with 75% alcohol before the next animal was tested. Each animal was tested twice and the average of the two trials was calculated for statistical analyses.

#### Rotarod

The Rotarod was used to assess the balance and motor coordination ability of mice. Three days before the test, the mice were placed on a Rotarod system (XR-YLS-4C, Shanghai, China) that set the speed at 20 rpm. The length of time that the mice stayed on the roller was recorded as the latency to fall, registered automatically by a trip switch on the floor of each rotating drum. The mice were trained for 3 min every day, lasting for three consecutive days.

#### Pole Test

To determine the degree of bradykinesia and ability to movement balance of PD mice, the pole test was conducted as previously described (Park et al., 2013). All the mice were pre-trained before MPTP injections. The height of the self-made simple climbing pole is 55cm, and the diameter of the top ball is 1 cm. The mice were placed on the top, and the time for the mice to turn from head up to head down was recorded as turn time (T-turn), and the time for the mice taken to arrive at the floor (locomotor activity time, T-LA) were recorded. If the mice remained motionless on the ball for more than 30 s, it was guided to head down, and T-turn was recorded as 30 s. Each animal was tested for three times and the average of the three trials was calculated for statistical analyses.

### Immunohistochemistry

Four mice per group were transcardially perfused with 0.9% NaCl and followed by 4% paraformaldehyde under anesthesia with isoflurane. Then, the brains were post-fixed in 4% paraformaldehyde for 24 h. The frozen brains were cut at 20 μm using a vibratome (Leica Microsystems, Wetzlar, Germany). Sections were permeabilized and blocked in PBS containing 0.3% Triton X-100 and 5–10% goat serum at room temperature for 1.5 h. Immunohistochemistry was performed on free-floating brain sections. Primary antibodies were: anti-TH (1:1000; Abcam, ab112); anti-GFAP (1:10; Abcam, ab4648); anti-IBA1(1:100; Abcam, ab178847). Primary antibodies were visualized with Alexa-Fluor 488 and Alex-Fluor 647 secondary antibodies. Images were acquired using an Olympus light microscope. For each animal of per group we randomly chose 5∼8 sections, and a total of 25∼40 sections were used for statistics in each group, there were four dots in the scatter plot, each dot in the scatter plot represents all the slices average value of each animal in each group. Fluorescence was quantified using Image-Pro Plus 6.0 by an experimenter blind to all groups.

### Transmission Electron Microscopy

Four mice per group were trans-cardially perfused with 0.9% NaCl and followed by 4% glutaraldehyde under anesthesia, Then the brains were removed and substantia nigra were dissected out. The tissue was fixed by 2.5% glutaraldehyde for 4 h and 1% osmic acid for 2 h, the gradient of dehydration was carried out using anhydrous ethanol and acetone. Ultrathin sectioning was performed on a microtome (LEICA EM UC7), the ultrathin sections were stained for 20 min in saturated uranyl acetate. Then, sections were rinsed and post-stained in lead citrate for 5 min, and were rinsed again and dried. The ultrastructures of sections were observed and imaged on a TEM (JEM-1400; Jeol Ltd., Tokyo, Japan) at 60 KV. For the observations of mitochondrial autophagosomes, five intact neurons at least were selected from each sample, then counted the total number of all mitochondria in the periplasm of each neuron under 30,000 multiples. For synapse observation, at least 15 fields were selected to count the synapse number from each sample under 30,000 multiples.

### Western Blotting

Four mice brain tissue per group was extracted from midbrain tissues containing substantia nigra, then samples were lysed in RIPA buffer (150 mM NaCl, 50 mM Tris-HCl pH 7.4, 0.1% SDS, 1% NP-40, 1% protein phosphatase inhibitor) on ice for 15 min. The samples were centrifuged at 14,000 rpm at 4°C for 15 min. Thereafter, lysates were quantitated using a nanodrop (SpectraMax iD5) and estimated total protein values (40–60 μg). Western blotting analysis was performed as described previously (Zhang et al., 2016). Primary antibodies were anti- synaptophysin (SYN; 1:10000; Abcam, ab32127), IL-1β(1:5000; Abcam, ab9722), ATG7(1:10000; Abcam, ab133528), Beclin1(1:2000; Abcam, ab207612), PGC1α (1:2000; Abcam, ab191838), Mfn2(1:1000; Abcam, ab124733), IRE1 (1:2500; Abcam, ab48187), Bcl-2(1:300; BOSTER, BM4985), Bax(1:1000; Abcam, ab32503), CREB^S133^ (1:500; Abcam, ab220798), CREB (1:500; Abcam, ab31387), and β-actin (1:400). Secondary antibodies were horseradish peroxidase-linked (GE Healthcare). Densitometric analysis was performed using ImageJ 1.46a and normalized to values of β-actin.

### Statistics

Data were analyzed using the program Prism7.0 (Graphpad software Inc., United States) and provided as means ± SEM, statistical significance were analyzed by Student’s t test, or one-way analysis of variance (ANOVA) followed by Bonferroni’s *post hoc* test. All data in the manuscript were performed for the normality of populations using the Shapiro Wilk test and homogeneity of variances before each ANOVA. Only results with *P* < 0.05 were considered statistically significant.

## Results

### Assessment of CCK Analogue Carboxyfluorescein Blood–Brain Barrier Penetration

**FIGURE 1:**
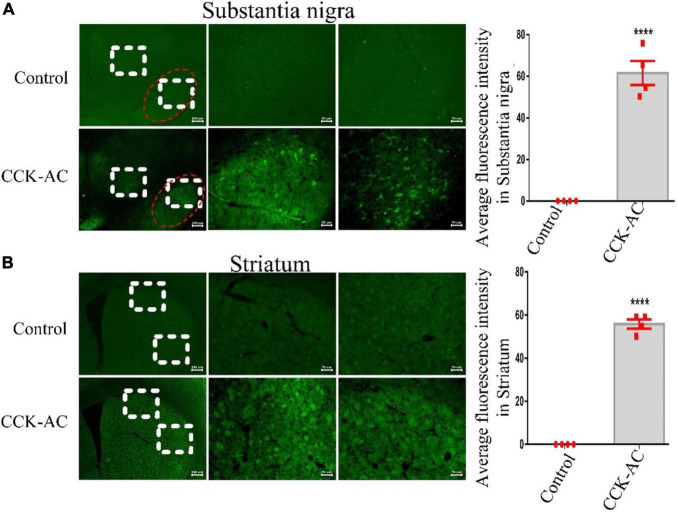
Assessment of CCK analogue for permeability of the BBB. **(A)** The expression of CCK-AC in the substantia nigra (The region of the red ellipse is substantia nigra pc. Scale bar: 200 μm, 50 μm). The expression of CCK-AC in the striatum (Scale bar: 200 μm, 50 μm). **(B)** A difference was found between two groups. ****P < 0.0001, compared with the control group, *n* = 4 per group.

In a Student’s t test, we found a difference between two groups in the substantia nigra (*P* < 0.0001) and striatum (*P* < 0.0001). The CCK analogue carboxyfluorescein can crossed the BBB and is abundantly expressed in the substantia nigra and striatum (Figures 1A,B).

### Both Liraglutide and the Cholecystokinin Analogue Alleviated 1-Methyl-4-Phenyl-1,2,3,6-Tetrahydropyridine-Induced Impairments in the Locomotor and Exploratory Activity of Mice

**FIGURE 2:**
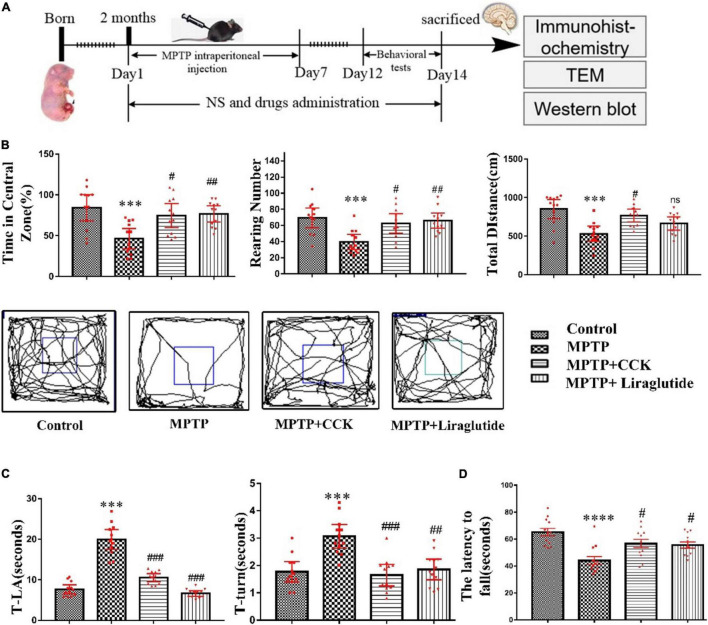
Liraglutide and the CCK analogue alleviated motor symptoms of MPTP mice. **(A)** The experimental design of our study. **(B)** Open-field locomotion test and movement trajectory diagram of mice in each group. **(C)** The results of the pole test. **(D)** Rotarod sensory motor performance of mice in each group. A difference was found between groups. *P < 0.05, ^**^P < 0.01, ^***^P < 0.001, ^****^P < 0.0001, compared with the control group, ^#^*P* < 0.05, ^##^*P* < 0.01, ^###^*P* < 0.001 and ns, compared with the MPTP group, *n* = 12 per group.

Our experimental design scheme is shown in Figure 2A. In a one-way ANOVA, an overall difference was found (*P* < 0.001) between the control group and MPTP group. *Post hoc* tests showed that, time in central zone (46.43 ± 5.598%), the total distance of movement (531 ± 44.66 cm) and the total numbers of rearing (39.5 ± 4.277) in the MPTP group, compared with the control group (84.19 ± 7.375%, *P* < 0.001; 852.3 ± 56.72 cm, *P* < 0.01; 69.33 ± 5.575, *P* < 0.001) exhibited a significant reduction. However, 2 weeks of treatment with liraglutide (time in central zone: 76.68 ± 4.475%, the total distance of movement: 664.7 ± 39.51 cm and the total numbers of rearing: 66.08 ± 4.22) or CCK analogue (time in central zone:% 74.64 ± 6.601, the total distance of movement: 769.1 ± 37.85 cm, and the total numbers of rearing: 62.5 ± 5.401) reversed both these effects on most of these indicators (*P* < 0.05, *P* < 0.01, *P* < 0.001, Figure 2B). However, liraglutide did not show a significant effect on the total distance of movement. According to the movement trajectory, it can be found that the range of activity of the MPTP group in the experimental group is gradually limited to the corner of the detection box.

In the pole test, we found an overall difference on T-turn and T-LA of mice between groups by a one-way ANOVA (*P* < 0.001). *Post hoc* tests showed that, compared with the control group (T-LA: 7.642 ± 0.507, T-turn: 1.767 ± 0.1689), MPTP injection induced the movement imbalance of mice, with more time required by mice to climb down (T-LA: 19.96 ± 1.11) (*P* < 0.001), and also induced bradykinesia of mice, with more time to turn at the top (T-turn: 3.067 ± 0.1959) (*P* < 0.001). However, treatment with liraglutide (T-LA: 6.633 ± 0.3187, T-turn: 1.854 ± 0.1716) and CCK analogue (T-LA: 10.53 ± 0.4568, T-turn: 1.646 ± 0.1787) significantly reversed these effects induced by MPTP (*P* < 0.01, *P* < 0.001, Figure 2C).

In the rotarod test, a one-way ANOVA found an overall difference between groups (*P* < 0.0001). The results showed that the impairments in muscular strength and movement balance of mice was induced after MPTP injection. Compared with the control group (65.18 ± 2.852), the MPTP group mice stayed less time on the rotating rod (the latency to fall) (44.05 ± 2.996) (*P* < 0.0001). However, treatment with liraglutide (55.59 ± 2.274) or the CCK analogue (56.67 ± 3.118) significantly reversed these effects induced by MPTP (*P* < 0.05, Figure 2D).

### Liraglutide and the Cholecystokinin Analogue Reversed Dopaminergic Neurons and Synapse Loss Induced by 1-Methyl-4-Phenyl-1,2,3,6-Tetrahydropyridine

**FIGURE 3:**
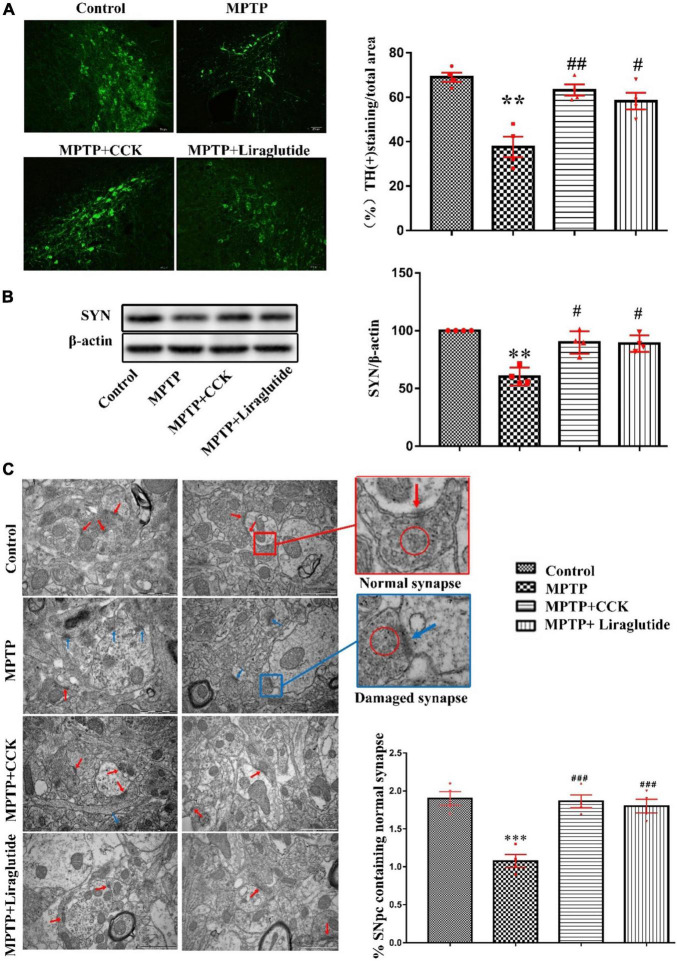
Liraglutide and the CCK analogue reversed dopaminergic neurons and synapse damage induced by MPTP. **(A)** The average area of TH-positive neurons in the SNpc of mice in each group (Scale bar: 50 μm). **(B)** Western blot showing the protein levels of SYN and actin as a loading control for each group. **(C)** TEM observation of synapse in the SNpc of mice in each group. Individual synapse were identified by the presence of at least three presynaptic vesicles (red circle) and an identifiable synaptic gap (normal synapse: red arrows and damaged synapse: blue arrows) (Scale bar: 1 μm). ^**^P < 0.01, ^***^P < 0.001, compared with the control group, ^#^*P* < 0.05, ^##^*P* < 0.01, ^###^*P* < 0.001, compared with the MPTP group, *n* = 4 per group.

A one-way ANOVA following by *post hoc* tests showed that, compared with the control group (69 ± 2.082%), the average area of Tyrosine hydroxylase (TH)-positive cells in the SNpc was reduced significantly after MPTP injection (37.63 ± 4.616%) (*P* < 0.01). However, liraglutide (58.25 ± 3.75%) and the CCK analogue (63.25 ± 2.496%) treatment restored the average area of dopaminergic neurons (*P* < 0.05, *P* < 0.01, Figure 3A). In addition, compared to the control group (100 ± 0), the marker for synapse, synaptophysin (SYN), was much reduced in mice treated with MPTP (60.37 ± 3.845) (*P* < 0.01), but liraglutide (88.83 ± 3.572) or CCK analogue (89.79 ± 4.898) treatment can reversed this reduction (*P* < 0.05, Figure 3B). Consistent with Western blotting results, the TEM analysis showed that, compared to the control group (1.9 ± 0.09129), the percentage that contains the normal number of synapses in the SNpc was significantly decreased in the MPTP group (1.075 ± 0.08539) (*P* < 0.001), the morphology showed that the synaptic gap was variable, and the length and width of synaptic active area and synaptic gap were significantly different (Figure 3C). However, treatment with liraglutide (1.8 ± 0.09129) and CCK analogue (1.863 ± 0.08509) significantly reversed these effects induced by MPTP.

### Liraglutide and the Cholecystokinin Analogue Decreased Glial Cells Activation and Neuroinflammation Induced by 1-Methyl-4-Phenyl-1,2,3,6-Tetrahydropyridine

**FIGURE 4:**
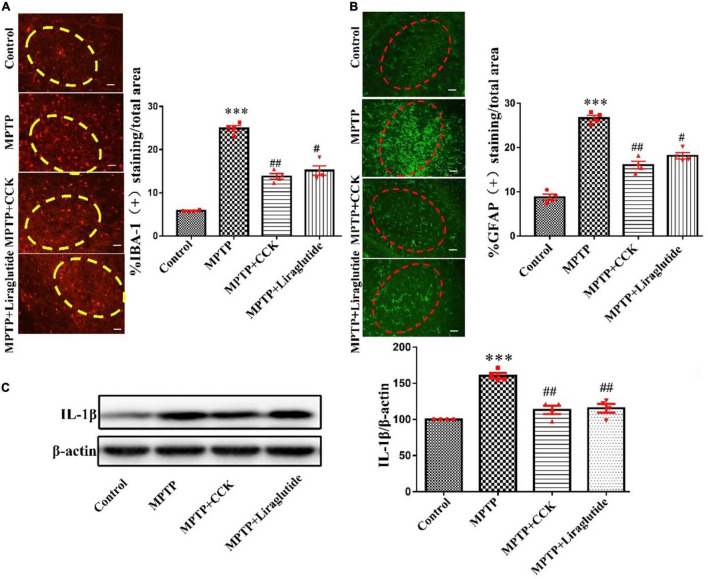
Liraglutide and the CCK analogue decreased microglia and astrocytes activation and the release of inflammatory cytokines induced by MPTP (The region of the ellipse is substantia nigra pc). **(A)** The expression of IBA-1 in the SNpc of mice in each group (scale bar: 50 μm). **(B)** The expression of GFAP in the SNpc of mice in each group (scale bar: 50 μm). **(C)** Expression of inflammatory cytokines IL-1β in the SNpc of mice in each group. ^***^P < 0.001, compared with the control group, ^#^*P* < 0.05, ^##^*P* < 0.01, compared with the MPTP group, *n* = 4 per group.

Immunohistochemistry was used to detect the expression of IBA-1 (Figure 4A), an microglial specific marker and GFAP (Figure 4B), an astrocyte specific marker in the SNpc of mice in each group. The results showed that, compared to the control group with IBA1-positive microglia covered an average of 6.35% of each section in the SNpc, the average area of IBA-1-positive cells in the MPTP group covered an average of 24.56% of each section increased significantly, the cell body was enlarged with more protuberances and branches (*P* < 0.001). While liraglutide and CCK analogue treatment made the areas of IBA1-positive microglia decrease to 14.8% (*P* < 0.01) and 16.3% (*P* < 0.05). Compared to the control group with GFAP-positive astrocyte covered an average of 6.38% of each section in the SNpc, the average area of GFAP-positive cells in the MPTP group covered an average of 27.86% of each section increased significantly (*P* < 0.001). While liraglutide and CCK analogue treatment made the areas of GFAP-positive astrocyte decrease to 17.8% (*P* < 0.01) and 18.9% (*P* < 0.05).

Activated glial cells secrete a large number of inflammatory cytokines. Western blotting was used to explore the levels of inflammatory cytokines IL-1β in the SNpc. Notably, compared with the control group (100 ± 0), the expression of IL-1β in the MPTP group (160.4 ± 3.845) was significantly increased (*P* < 0.001), after liraglutide (115.5 ± 6.013) and CCK analogue (113.1 ± 5.635) injection, the protein expression of IL-1β also decreased in contrast to the MPTP group (*P* < 0.01) (Figure 4C).

### Liraglutide and the Cholecystokinin Analogue Regulated Autophagy Dysfunction Induced by 1-Methyl-4-Phenyl-1,2,3,6-Tetrahydropyridine

**FIGURE 5:**
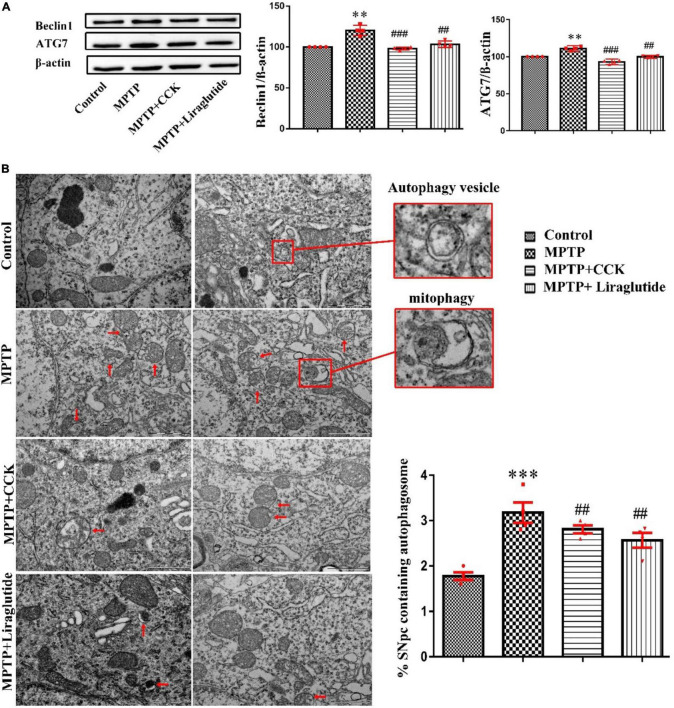
Liraglutide and the CCK analogue regulated autophagy dysfunction induced by MPTP. **(A)** Western blot showing the protein levels of ATG7, Beclin1 and actin as a loading control for each group. **(B)** TEM observation of autophagosomes (autophagic vesicles and mitophagy, red arrows) in the SNpc of mice in each group. Autophagy dysfunction induced by MPTP can be alleviated by liraglutide and CCK analogue treatment (Scale bar: 1 μm). ^**^P < 0.01, ^***^P < 0.001, compared with the control group, ^##^*P* < 0.01, ^###^*P* < 0.001, compared with the MPTP group, *n* = 4 per group.

Western blotting was used to explore the levels of autophagy protein ATG7 and Beclin1 in the SNpc of mice in each group (Figure 5A). Notably, compared with the control group (100 ± 0), the expression of ATG7(111.1 ± 1.255) and Beclin1(120.4 ± 3.111) in the MPTP group was significantly increased (*P* < 0.01), after liraglutide or CCK analogue injection, the protein expression of ATG7(liraglutide: 99.8 ± 0.6613, CCK: 93.01 ± 1.174) and Beclin1(liraglutide: 103.4 ± 1.948,CCK: 98.08 ± 0.8579) also decreased in contrast to the MPTP group (*P* < 0.01, *P* < 0.001).

Transmission Electron Microscopy is the “gold standard” for the observation of autophagy. Consistent with findings from western blotting, TEM further demonstrated that autophagy dysfunction was induced by MPTP (3.175 ± 0.225) (*P* < 0.001) compared with the control group (1.775 ± 0.08539), which is alleviated by liraglutide (2.566 ± 0.1619) and CCK analogue (2.808 ± 0.09069) treatment. The mitochondrial structure was disordered, and some of them were wrapped into vesicles, known as mitophagy. There were few mitophagy vesicles after liraglutide and CCK analogue injection comparing with the MPTP group (*P* < 0.01, Figure 5B).

### Liraglutide and Cholecystokinin Analogue Protected Against Mitochondrial Damage and ER Stress Induced by 1-Methyl-4-Phenyl-1,2,3,6-Tetrahydropyridine

**FIGURE 6:**
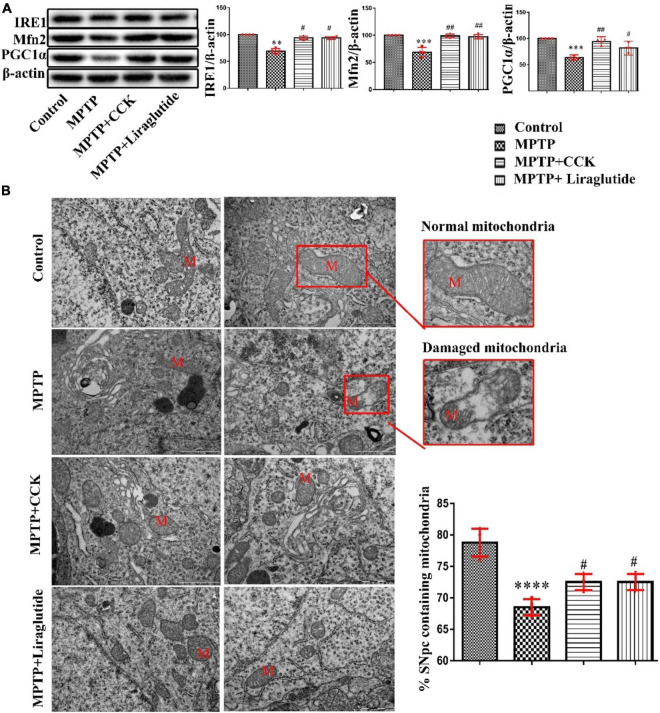
Liraglutide and CCK analogue protected against mitochondrial damage and ER stress induced by MPTP. **(A)** Western blot showing the protein levels of PGC1α, Mfn2, IRE1 and actin as a loading control for each group. **(B)** TEM observation of mitochondria (M) in the SNpc of mice in each group (Scale bar: 1 μm). ^**^P < 0.01, ^***^P < 0.001, ^****^P < 0.0001 compared with the control group, ^#^*P* < 0.05, ^##^*P* < 0.01, compared with the MPTP group, *n* = 4 per group.

Western blotting was used to explore the levels of mitochondrial biosynthesis related proteins PGC1α and Mfn2 in the SNpc of mice in each group (Figure 6A). Notably, compared with the control group (100%), the expression of PGC1α (63.3 ± 1.71) and Mfn2(68.95 ± 4.271) in the MPTP group was significantly decreased (*P* < 0.001), after liraglutide and CCK analogue injection, the protein expression of PGC1α (liraglutide: 81.67 ± 4.142, CCK: 94.43 ± 2.737) and Mfn2(liraglutide: 97.28 ± 1.999, CCK: 98.78 ± 1.757) also increased in comparison with the MPTP group (*P* < 0.05, *P* < 0.01). IRE1 is the core receptor of ER stress. As shown in Figure 6A, compared with the control group (100%), the expression level of IRE1 was decreased by MPTP injection (69.5 ± 2.359) (*P* < 0.01), liraglutide (93.71 ± 1.282) and CCK analogue (93.96 ± 1.588) treatment reversed the decrease of IRE1 significantly (*P* < 0.05).

Consistent with findings from western blotting, TEM further confirmed that the structure and morphology of mitochondria in the control group were intact. In contrast, in the MPTP group, a large number of mitochondria with round, swollen, sparse cristae, and even vesicular degeneration were seen. Compared to the control group (78.75 ± 1.109), the percentage that contains the normal number of synapses in the SNpc was significantly decreased in the MPTP group (68.5 ± 0.6455), Liraglutide (72.03 ± 0.6455) and CCK analogue (71.98 ± 0.5643) treatment inhibited mitochondrial damage significantly (Figure 6B).

### Liraglutide and the Cholecystokinin Analogue Protected Against Apoptotic Signaling and the Decrease of pCREB^S133^ Induced by 1-Methyl-4-Phenyl-1,2,3,6-Tetrahydropyridine

**FIGURE 7:**
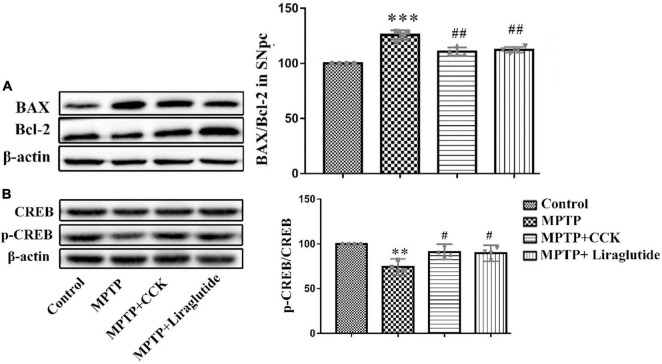
Western blot showing the protein levels of Bax, Bcl-2 and CREB. Liraglutide and the CCK analogue reversed the increase of ratio of Bax/Bcl-2 **(A)** and the decrease of pCREB^S133^ **(B)** in the SNpc of mice induced by MPTP. ^**^P < 0.01, ^***^P < 0.001, compared with the control group, ^#^*P* < 0.05, ^##^*P* < 0.01, compared with the MPTP group, *n* = 4 per group.

Western blotting was used to explore the levels of anti-apoptotic signaling molecule B-cell lymphoma-2 (Bcl-2) and pro-apoptotic signaling molecule BCL2-Associated X (Bax) in the SNpc of mice in each group. Compared with the control group (100%), the ratio of Bax/Bcl-2 was increased in the MPTP group (125.8 ± 2.11) (*P* < 0.001), while the ratio of Bax/Bcl-2 was partly decreased by enhancing Bcl-2 levels after liraglutide (112.3 ± 1.325) and CCK analogue (110.7 ± 1.845) treatment (*P* < 0.01) (Figure 7A).

There was no change about the levels of total CREB in any of the groups. As shown in Figure 7B, compared with the control group (100%), the expression level of pCREB^S133^ was decreased by MPTP injection (74.3 ± 2.873) (*P* < 0.01), liraglutide (89.67 ± 2.825) and CCK analogue (91.09 ± 2.698) treatment reversed the decrease of pCREB^S133^ significantly (*P* < 0.05).

## Discussion

In this study we demonstrate for the first time that CCK receptor activation has neuroprotective effects in an animal model of PD. As animals do not naturally develop PD, several animal models of PD are used in experimental studies, such as 6-hydroxydopamine (6-OHDA) rat model, MPTP mouse model or the A53T transgenic mouse model. The MPTP lesion model is one of several models that are in use to investigate the underlying mechanisms of PD and also to test new drug candidates for their neuroprotective properties (Morin et al., 2014). MPTP can cross the blood-brain barrier, then it is metabolized into 1-methyl-4-phenylpyridinium (MPP +) by the monoamine oxidase B in astrocytes (Glover et al., 1986). The electron-transport chain of mitochondria is the main site of intracellular ROS production (Sangwung et al., 2020). MPP + is taken up by dopaminergic neurons and interferes with normal electron transfer by inhibiting the activity of mitochondrial respiratory chain complex I, which causes the increase of ROS levels and resulting mitochondrial damage, eventually leading to dopamine neuronal injury and death in the SNpc, and producing the symptoms of PD (Nakamura and Vincent, 1986; Gerlach et al., 1991; Hu et al., 2015). In our study, open field test analysis suggests that liraglutide and the CCK analogue both improved the locomotor and exploratory activity of mice induced by MPTP, in the pole test and rotarod test, two drugs had the same effect on improving bradykinesia, movement coordination and balance of mice. There was no significant difference between the two drugs in improving symptoms of mice induced by MPTP. The impairment in locomotor performance was consistent with the reduced levels of TH protein levels, which is a key enzyme in the dopamine synthesis. We estimated the average area covered by each cell population for TH in the SNpc after acute MPTP injection, and found that both liraglutide and the CCK analogue treatment restored the average area of TH-positive neurons reduced by MPTP. Neuronal damage leads to structural changes and a decrease in the number of synapses. Furthermore, as shown in the results of TEM and synapse biomarker synaptophysin analysis, the synapse numbers are reduced by the MPTP treatment, and liraglutide and the CCK analogue were able to partially prevent or reverse this effect. These results demonstrate protective effects of liraglutide and CCK analogue on synapses and synaptic function in the MPTP treated mice.

The loss of neurons is the key to the pathophysiological process of neurological diseases, which is mediated by oxidative stress, mitochondrial dysfunction, abnormal protein aggregation and other factors (Saiki et al., 2012; Segura-Aguilar and Kostrzewa, 2015). One of the most important factors is oxidative stress (Bisaglia et al., 2013; Toulorge et al., 2016; Loeffler et al., 2017). MPTP can cause mitochondrial damage and the increase of ROS, which aggravates mitochondrial damage. The main biological feature of PGC1α gene is to stimulate mitochondrial biosynthesis, thus replacing damaged mitochondria and improving the ability of cells to produce ATP (Pacelli et al., 2011). Mitofusin2 (Mfn2) regulates mitochondrial fusion, transport and autophagy. IRE1 is the core receptor of ER stress, which can sense the accumulation of unfolded proteins in the endoplasmic reticulum lumen and transmit this stimulation to other areas of the cell. Liraglutide and CCK analogue increased the levels of PGC-1α, Mfn2 and IRE1 in the SNpc decreased by MPTP, thus reducing the oxidative stress response of mitochondria and enhancing mitogenesis. TEM analysis also confirmed that the morphological structure and number of mitochondria had partially recovered.

Chronic inflammation is a key factor in the progression of PD. ROS can participate in many physiological processes, including apoptosis and the inflammatory response (Dionisio et al., 2019). The main characteristic of neuroinflammation is the activation of glial cells (Alam et al., 2016). In this study, we detected the expression of astrocyte specific marker GFAP and microglial specific marker IBA – 1 in the SNpc of mice, the results showed that they increased in MPTP mice significantly. Activated glial cells can produce a variety of inflammatory cytokines, some inflammatory cytokines, such as IL-1β (Huang et al., 2005). We detected the protein expression of IL-1β, which was significantly increased in MPTP mice, and it can be reduced by liraglutide and CCK analogue treatment. The reduction of the inflammation response is most likely one of the mechanisms by which CCK analogue exert their neuroprotective effects.

Inflammation and autophagy interact in the brain of PD patients (Jones et al., 2013; Netea-Maier et al., 2016). In addition, appropriately increased ROS levels can activate mitophagy, which is conducive to cell survival, while excessive ROS will be detrimental (Lin and Kuang, 2014; Filomeni et al., 2015). On the one hand, autophagy is considered to be a protective mechanism against ROS by removing harmful components to prevent oxidative damage (Meng et al., 2013). In particular, selective elimination of dysfunctional mitochondria through autophagy plays a protective role, limiting ROS production and preventing potential oxidative damage. On the other hand, if ROS levels exceed the scavenging capacity of autophagy, damaged mitochondria and misfolded proteins such as alpha-synuclein continue to build up, and neuronal death will occur. Oxidative stress and mitophagy defects are considered to be the main causes of dopaminergic neuronal death in the SNpc of PD patients (Menzies et al., 2017; Miki et al., 2018). Liraglutide and the CCK analogue reduced excessive autophagy induced by MPTP, and restored the function of organelles, which was also confirmed by TEM.

A balanced ratio between the pro-apoptotic signaling molecule Bax and the anti-apoptotic signaling molecule Bcl-2 can maintain normal function of mitochondria. When the Bax level is increased and the Bcl-2 level is decreased, apoptosis is triggered (Wu et al., 2019). MPTP has been shown to induce apoptosis by inhibiting the mitochondrial multi-subunit enzyme complex I (Bove and Perier, 2012; Konnova and Swanberg, 2018). We found a decline of Bcl-2 levels and an increase of Bax after MPTP treatment, and liraglutide and CCK analogue partly reversed this by promoting Bcl-2 expression. TEM also confirmed that liraglutide and CCK analogue decreased apoptosis.

The peptide growth factors and hormones liraglutide and CCK analogue all activate a classic 7-membrane spanning, G-protein coupled receptor that activates adenylylcyclase and increases cAMP levels in the cell, which in turn activates Protein Kinase A (PKA) and phosphorylates the cAMP-response element binding protein (CREB) at Serine 133 (Mayo et al., 2003; Lee and Soltesz, 2011; Rehfeld, 2017). It has been established for a long time that cAMP is protective in cells, so it is not surprising that all of these peptide growth hormones have neuroprotective properties (Cameron and Kapiloff, 2017). In addition, the cAMP-CREB transcription pathway is important for synaptic plasticity and memory formation (Nazif et al., 1991; Bernabeu et al., 1996). We therefore tested the levels of CREB phosphorylation at serine 133 in the SNpc. There was a decline in levels of p-CREBS^133^ after MPTP treatment. However, the CCK analogue treatment partially reversed the decrease of p-CREB^S133^ levels. These findings demonstrate that the CCK analogue can facilitate CREB activation, and ultimately promote cell survival of dopaminergic neuron in SNpc. It has been reported that liraglutide can promote the expression of brain-derived neurotrophic factor (BNDF; de Souza et al., 2019; Chaves Filho et al., 2020), importantly, the expression of BNDF can be enhanced by CREB signaling activation (Zhen et al., 2016). While autophagy is activated by growth factor cell signaling, eg., *via* Akt activation which in turn activates mTOR, one of the key activators of autophagy (Heras-Sandoval et al., 2014; Moulis and Vindis, 2017). Therefore, the neuroprotective mechanism by which neurons are rescued is most likely linked to the enhanced p-CREB^S133^ levels, which in turn promote the expression of genes of growth factors such as BDNF.

The MPTP mouse model is considered as an animal model of PD, but it still has certain limitations and cannot summarize all the pathological features of PD. Our results suggest that CCK analogue may be a potential treatment for PD. Further experiments can be performed using other PD models such as 6-OHDA or LPS injection into the brain, or an alpha-synuclein mutation expressing transgenic mouse model.

In conclusion, the results from this PD mouse model study demonstrate for the first time that CCK signaling has neuroprotective effect that are equal to those of GLP-1 signaling, see Hölscher (2020) for a review. As the GLP-1 receptor agonist exendin-4 has already shown good neuroprotective effects in a phase II clinical trial in PD patients (Athauda et al., 2017, 2019), it is of great interest to demonstrate that a different peptide hormone from the group of metabolic signaling peptides can show similar neuroprotective properties. Further research is required to profile the neuroprotective effects of CCK in more detail.

However, the current study demonstrates the CCK holds promise as novel treatments for neurodegenerative processes that not only improve the symptoms but prevent the neurodegenerative processes underlying PD.

In addition, a key question that is emerging is whether CCK analog and Litraglutide have an additive effect. Next, CCK/GLP-1 dual-agonist can be designed to treat Parkinson’s disease. This kind of dual-agonist not only activates the CCK receptor but also the GLP-1 receptor. Similar dual agonists have been developed to treat diabetes nowadays. The activation of both CCK and GLP-1 receptors may be a superior strategy for protecting neurons from damage.

## Data Availability Statement

The original contributions presented in the study are included in the article/Supplementary Material, further inquiries can be directed to the corresponding author/s.

## Ethics Statement

All procedures involved in this study were approved by the Animal Care and Use Committee of Henan University of Chinese Medicine (No: DWLL201903076).

## Author Contributions

SS, MS, YY, ZY, HL, YS, and JM conducted the work. ZJZ, ZQZ, and CH supervised the work and wrote the manuscript. All authors contributed to the article and approved the submitted version.

## Conflict of Interest

The authors declare that the research was conducted in the absence of any commercial or financial relationships that could be construed as a potential conflict of interest.

## Publisher’s Note

All claims expressed in this article are solely those of the authors and do not necessarily represent those of their affiliated organizations, or those of the publisher, the editors and the reviewers. Any product that may be evaluated in this article, or claim that may be made by its manufacturer, is not guaranteed or endorsed by the publisher.
