# Supplementary material for: Neuroprotective Effects of a Cholecystokinin Analogue in the 1-Methyl-4-Phenyl-1,2,3,6-Tetrahydropyridine Parkinson’s Disease Mouse Model
Source: Front Neurosci. 2022 Mar 15;16:814430. doi: 10.3389/fnins.2022.814430 (PMC8964967; doi:10.3389/fnins.2022.814430)

**The expression of fluorescence signals in the cortex, hippocampus,** **Substantia nigra and Striatum region (Scale bar:** **200μm,** **50μm)**


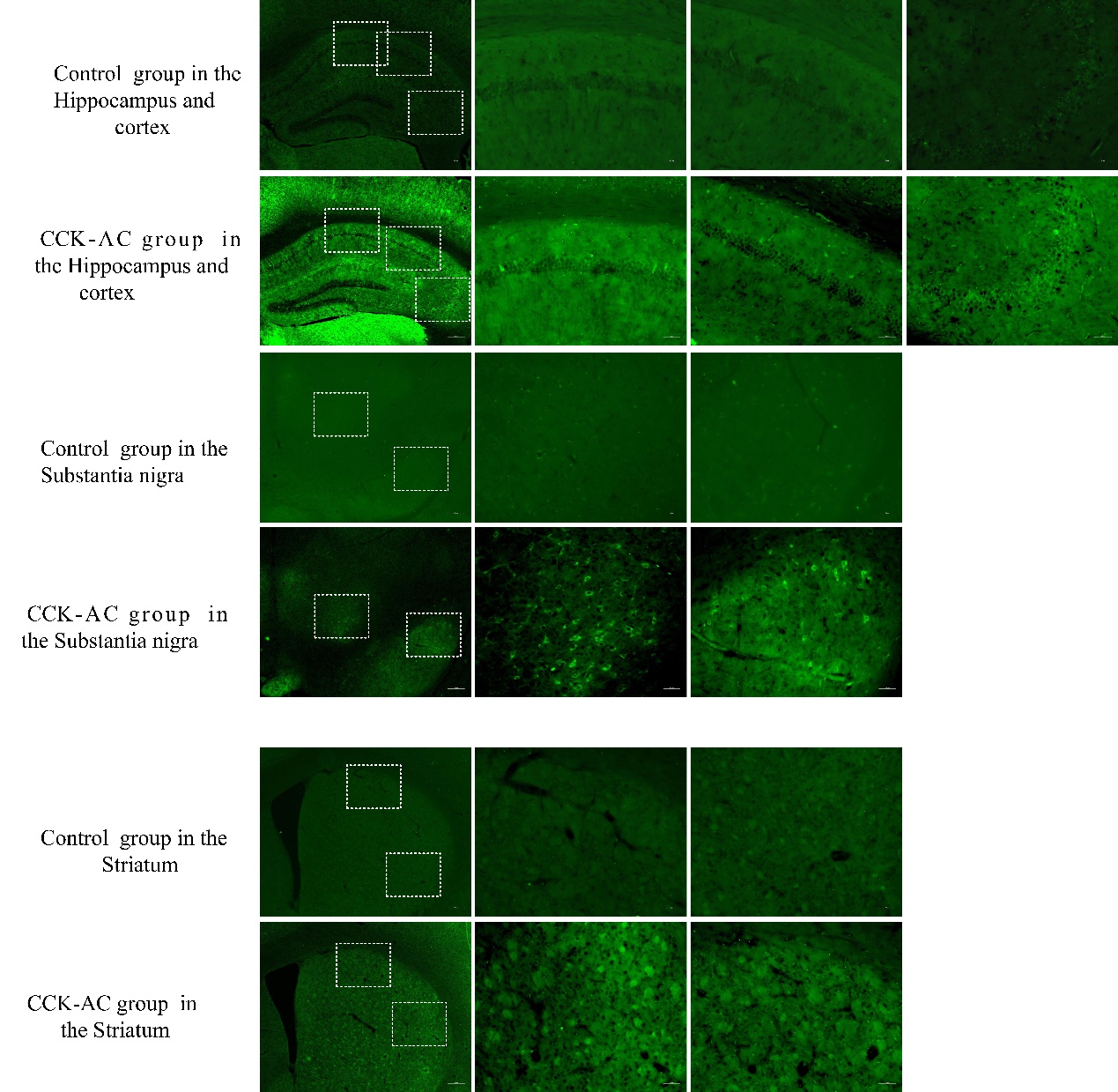


**Western blot**

Fig4：

IL-1β


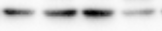

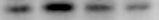

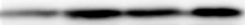


Fig5：

ATG7


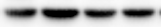

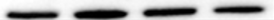

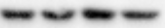


Beclin1


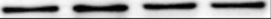

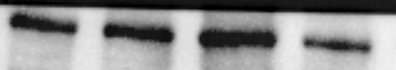

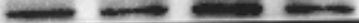


Fig6：

IRE1


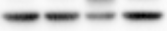

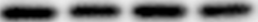

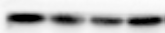


Mfn2


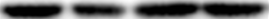

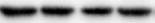

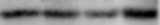


PGC1α


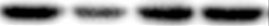

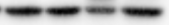

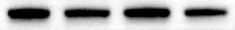


Fig7：

Bcl-2


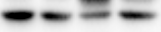

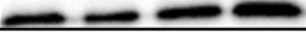

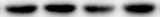


BAX


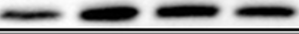

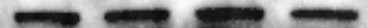

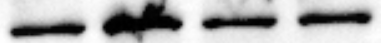

Supplement: Supplementary file 1 [file Table_1.DOCX]
